# The effects of dairy and dairy derivatives on the gut microbiota: a systematic literature review

**DOI:** 10.1080/19490976.2020.1799533

**Published:** 2020-08-23

**Authors:** Hajara Aslam, Wolfgang Marx, Tetyana Rocks, Amy Loughman, Vinoomika Chandrasekaran, Anu Ruusunen, Samantha L. Dawson, Madeline West, Eva Mullarkey, Julie A. Pasco, Felice N. Jacka

**Affiliations:** a IMPACT - The Institute for Mental and Physical Health and Clinical Translation, School of Medicine, Barwon Health, Deakin University, Geelong, Victoria, Australia; b Department of Psychiatry, Kuopio University Hospital, Kuopio, Finland; c Institute of Public Health and Clinical Nutrition, University of Eastern Finland, Kuopio, Finland; d Environmental & Genetic Epidemiology Research, Murdoch Children’s Research Institute, Royal Children’s Hospital, Parkville, Australia; e Psychology Department, Wellesley College, Wellesley, MA, USA; f Department of Medicine – Western Health, Melbourne Medical School, The University of Melbourne, St Albans, Victoria, Australia; g Department of Epidemiology and Preventive Medicine, Monash University, Prahran, Victoria, Australia; h Barwon Health, Geelong, Victoria, Australia; i Department of Psychiatry, University of Melbourne, Victoria, Australia; j Centre for Adolescent Health, Murdoch Children’s Research Institute, Victoria, Australia

**Keywords:** Gut microbiota, dairy, inflammation, casein, whey, bio-active peptide

## Abstract

The effects of dairy and dairy-derived products on the human gut microbiota remains understudied. A systematic literature search was conducted using Medline, CINAHL, Embase, Scopus, and PubMed databases with the aim of collating evidence on the intakes of all types of dairy and their effects on the gut microbiota in adults. Risk of bias was assessed using the Cochrane risk-of-bias tool.The search resulted in 6,592 studies, of which eight randomized controlled trials (RCTs) met pre-determined eligibility criteria for inclusion, consisting of a total of 468 participants. Seven studies assessed the effect of type of dairy (milk, yogurt, and kefir) and dairy derivatives (whey and casein) on the gut microbiota, and one study assessed the effect of the quantity of dairy (high dairy vs low dairy). Three studies showed that dairy types consumed (milk, yogurt, and kefir) increased the abundance of beneficial genera *Lactobacillus* and *Bifidobacterium*. One study showed that yogurt reduced the abundance of *Bacteroides fragilis*, a pathogenic strain. Whey and casein isolates and the quantity of dairy consumed did not prompt changes to the gut microbiota composition. All but one study reported no changes to bacterial diversity in response to dairy interventions and one study reported reduction in bacterial diversity in response to milk intake.In conclusion, the results of this review suggest that dairy products such as milk, yogurt, and kefir may modulate the gut microbiota composition in favor to the host. However, the broader health implications of these findings remain unclear and warrant further studies.

## Introduction

Dairy and dairy-derived products are a common component of many diets and influence physiological functions.^1-4^ Dairy products, including milk, yogurt and cheese, are generally considered nutrient-dense foods that contain proteins, calcium, and other essential nutrients such as magnesium, potassium, phosphorus, zinc, and B vitamins and their intake is associated with higher diet quality.^5,6^ The health properties of dairy foods have been the focus of intense research interest, evident from the substantial volume of published studies investigating the associations between dairy consumption and diseases such as cardiovascular disease (CVD), type 2 diabetes mellitus (T2DM), obesity, and osteoporosis.^1,7-18^ However, the influence of dairy consumption on health outcomes is equivocal and often controversial, with some studies suggesting a detrimental impact on health outcomes such as fractures^19^ and even clinical depression^20^ and both positive and inverse associations observed between dairy intake and CVD,^7-10^ T2DM^11-14^ and obesity.^15,16^ This may be largely due to the complex nutritional composition of dairy products, which also poses challenges in understanding the underlying mechanism driving these observed associations.

The majority of the health impacts of dairy may be related to its nutritional composition and caloric content, however, dairy products may also influence health outcomes via the gut microbiota. It is increasingly understood that modulating the gut microbiota is a key pathway by which dietary intakes may influence health outcomes.^21^ The gut microbiota is a dynamic metabolic organ that exerts diverse functions both localized to the gut and extending to peripheral organs.^22,23^ Diet in conjunction with other factors such as host genetics, age, sex and medication use, influences the structure and function of the gut microbiota.^22,24-27^ Although there is emerging evidence that overall diet quality^28,29^ and individual macronutrients and micronutrients of diet have a role in influencing the gut microbiota composition,^24,30^ the particular influence of dairy consumption on the gut microbiota composition is yet unclear.

Generally, a total of 3–4 serves/day of dairy products is recommended for adults, although this amount may vary depending on age, sex, and other physical requirements.^31,32^ Consumers in many countries can access a wide range of dairy products, including milk with varying fat content and fortified with additional vitamins and minerals, fermented milk/dairy (e.g. kefir, yogurt, cheese) and milk/dairy derivatives (e.g. casein, whey). Each of these products possesses distinct biological profiles and nutrient characteristics that may differentially influence the gut microbiota composition. Evidence from animal studies show that components of milk (e.g. fat^33^ and protein^34^) and dairy derivatives (e.g. casein and whey isolates^35^) can prompt compositional changes to the gut microbiota, whilst there is some limited evidence in humans to show the impacts of some dairy groups (e.g. yogurt, acidified milk) on the gut microbiota.^36^ Therefore, we aimed to undertake a comprehensive systematic literature review of extant intervention studies examining the influence of type of dairy products and dairy derivatives, including the quantity consumed, on human gut microbiota composition. The primary aim of our study was to investigate changes in the gut microbiota composition, measured in feces, in terms of relative abundance, colony forming units (CFU), or bacterial diversity (alpha and beta diversity) in response to dairy product consumption. Also, we further elaborated on mechanistic pathways by which dairy products may influence the gut microbiota composition.

## Methods

### Literature search

This review was written in accordance with the PRISMA (Preferred Reporting Items for Systematic reviews and Meta-Analyzes) statement^37^ and was registered on PROSPERO (CRD42019137318). A search strategy was developed based on the research question (Table 1). Studies were identified using the following databases: Medline, CINAHL, Embase, Scopus and PubMed. Medical subject headings (MeSH) terms used in the search were related to milk and milk derived products (bovine milk OR cow* milk OR milk product* OR dairy product* OR cultured milk product* OR cheese OR yogurt OR kefir OR fermented dairy OR milk cream OR casein OR casein isolates OR casein concentrates OR whey OR whey isolates OR whey concentrates OR beta-casein) and gut microbiota (gut microb* OR fecal microb* OR gastrointestinal microbiome OR 16sRNA sequencing OR meta-genomics). The search strategy identified articles published since journal inception up to February 2019.

**Table 1.** PICOS criteria for inclusion.

Parameter	Criteria
Population	Human participants, both clinical (diseased e.g. CVD, T2DM) and healthy
Intervention	Bovine/cow’s milk Fermented/cultured dairy Casein/whey isolates Milk protein supplements
Comparator	Other proteins (e.g. soy, meat) Other milk types (sheep, goat, donkey and human) Standard diet No dairy
Outcomes	Gut microbiota composition assessed from faeces

CVD: cardiovascular disease; T2DM: diabetes 2 mellitus.

The inclusion criteria were: randomized controlled trials (RCTs), including cross-over studies; RCTs that included both healthy and diseased subjects (clinical); published in English; included cow’s milk/milk-derived products as intervention (e.g. fermented or non-fermented dairy, whey protein, and milk protein supplements); and gut microbiota as the primary or secondary outcome (Table 1). Plant proteins, animal proteins, other sources of milk (sheep, goat, donkey, and human), and standard diets were considered as the comparator. Controls with no intervention/placebo were also considered as comparators. Additionally, studies that compared the effects of the quantity of dairy consumed were included. However, studies that examined the effects of non-dairy ingredients (e.g. prebiotic and probiotic supplement) that used dairy products as a medium were excluded (e.g. a yogurt enriched with a prebiotics/probiotics vs yogurt without prebiotic/probiotic supplement). Studies that investigated the changes in the gut microbiota composition (i.e. relative abundance, CFU, bacterial diversity) using any analysis techniques such as 16S rRNA, meta-genomics, qPCR, and culture dependant techniques were qualified to be included.

### Data extraction

Titles and/or abstracts of studies were retrieved using the search strategy and two independent review authors (HA, VC) identified studies that met the inclusion criteria. The full texts of these potentially eligible studies were retrieved and independently assessed by two authors. Any disagreement was resolved through discussion with a third reviewer (WM, TR, AL).

A standardized, piloted form was used to extract data from the included studies for the assessment of study quality and evidence synthesis. Extracted study information included: study design; setting, sample size; study period; participant characteristics (age, sex, comorbidities); details of the intervention and comparator (quantity, duration); changes in the gut microbiota (CFU, relative abundance, single species, alpha, or beta diversity) and time points of assessment; and information for assessment of risk of bias. Four authors (HA, SD, MW, EM) extracted data independently and discrepancies identified were resolved through discussion with a fifth author (WM).

### Risk of bias assessment

Risk of bias was assessed by four independent authors (HA, SD, MW, EM) using the Cochrane risk-of-bias tool for randomized controlled trials.^38^ This is a 5-domain tool with signaling questions that assesses the risk of bias due to randomization, deviations from intended interventions, missing outcome data, measurement of the outcome, and selection of reported results. The signaling questions in each domain will assist in judging the risk of bias in the relevant domain (i.e. high risk, low risk and some concerns) with the aid of a scoring algorithm. Finally, the overall risk of bias of the study was judged as low risk of bias, some concerns, or high risk of bias based on the judgment received in each individual domain. Conflicting judgment for studies were resolved collaboratively.

## Study results

**Figure 1.**
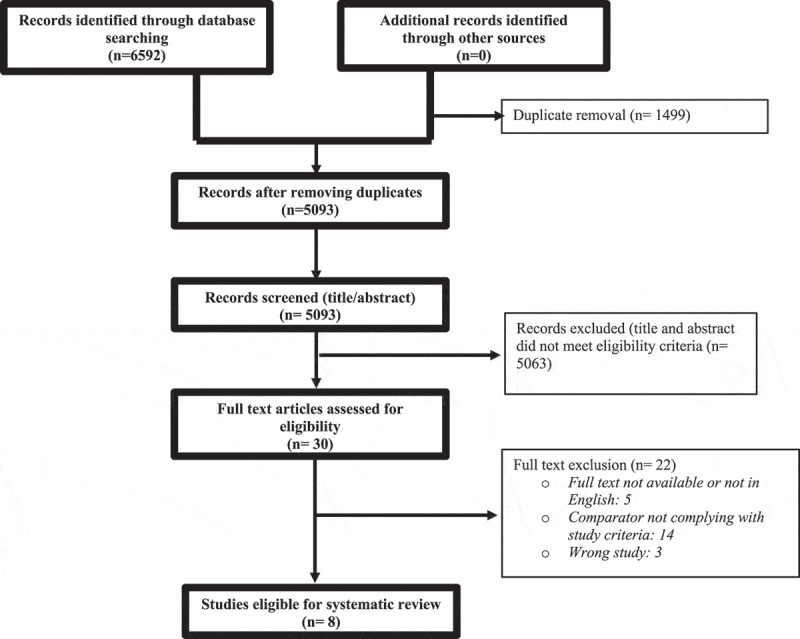
Prisma flow diagram.

As represented in Figure 1, the search strategy resulted in 5093 de-duplicated studies that were screened to identify eight eligible studies for inclusion.

### Study characteristics

A total of 468 participants were enrolled in the included studies. Trials ran for between 3 and 24 weeks with 12 weeks being the most common trial length (n = 3/8). Of the eight studies included, seven assessed the effect of dairy type (milk, yogurt, and kefir) and dairy derivatives (whey and casein) on the gut microbiota,^39-45^ and one study assessed the effect of consumed dairy quantity (high dairy vs low dairy) on the gut microbiota.^46^ The mean age of the participants was 39 years (range 32–44 years). Seven out of eight studies included both males and females^40-46^ with one study including only males.^39^ Studies were conducted in several countries including Switzerland, France, Denmark, Turkey, Japan, United States of America, and Canada (Table 2). Two studies were led by the same researcher.^41,42^ Risk of bias varied across studies: two studies were considered low risk of bias,^44,45^ five studies were considered to have some concerns,^39,41-43,46^ and one study had a very high risk of bias.^40^

**Table 2.** Population characteristics.

Study	Country	Sample size enrolled and completed	Female%	Age mean	Population type	Inclusion criteria
Fernandez-Raudales et al.^39^	United Sates of America	Enrolled: 81 Completed: 64	0	32	Overweight subjects	(i) No usage of antibiotics; (ii) Non-lactose intolerant;(iii) No smoking;(iv) Non-athlete; (v) Non-vegetarian
Link-Amster et al.^40^	Switzerland	Enrolled: 30 Completed: 30	14	47	Healthy	(i) Non-lactose intolerant; (ii) No recent antibiotic treatment; (iii) No vaccination with *Salmonella typhi* during 5 year period preceding the study
Odamaki et al.^41^	Japan	Enrolled: 33 Completed: 31	68	52	Healthy	(i) No vegetarians; (ii) Normal stool frequency
Odamaki et al.^42^	Japan	Enrolled: 32 Completed: 32	66	40	Healthy	(i) Consumption of yogurt less than twice a week
Yilmaz et al.^43^	Turkey	Enrolled: 45 Completed: 45	49	39	Patients with IBD	(i) No allergies or intolerance to milk; (ii) Alcohol consumption < 20 g/day; (iii) No antibiotic treatment within the last 1 month; (iv) No column or bowel operation history up to 3 months before the start of the study; (v) No infection in proceeding month
Beaumount et al.^44^	France	Enrolled: 42 Completed: 38	66	30	Healthy overweight (25< BMI <30)	(i) No GI disease; (ii) No usage of antibiotics during the last 3 months before the intervention; (iii) No dairy intolerance; (iv) No smoking
Reimer et al.^45^	Canada	Enrolled: 125 Completed: 96	53	40	Overweight or obese (BMI > 25, age 18–75)	(i) No antibiotics during the past three months; (ii) Body mass <350 Ib; (iii) No major GI surgeries, diabetes, CVD, liver pancreas disease; (iv) No chronic use of antacids or bulk laxative
Bendtsen et al.^46^	Denmark	Enrolled: 80 Completed:52	87	44	Overweight subjects	(i) No gastrointestinal diseases; (ii) No dairy food allergies; (iii) No infectious or metabolic diseases; (iv) No use of dietary supplements during the study or 6 months prior to the study; (iv) No use of cholesterol-lowering medicine; (vii) Women could not be pregnant or lactating

BMI: body mass index; CVD: cardiovascular disease; GI: gastrointestinal; IBD: inflammatory bowel disease.

### Interventions

Of the seven studies that assessed the effect of various types of dairy and/or dairy derivatives, four assessed the effect of fermented dairy including yogurt and kefir,^40-43^ two assessed the effect of whey and casein proteins,^44,45^ and one assessed the effect of bovine milk.^39^ Only one study investigated the quantity of dairy (low vs high intake) consumed.^46^

### Microbiome quantification

Two studies used quantitative polymerase chain reaction (qPCR)^42,43^ and four used 16S rRNA sequencing.^41,44-46^ The seventh study used both qPCR and bacterial Tag-encoded FLX amplicon pyrosequencing (bTEFAP) sequencing.^39^ A culture dependant method was used by another study by Link-Amster et al.^40^ (Table 3).

**Table 3.** Methodological characteristics of articles reviewed.

Study	Study design	Trial duration	Sample size calculation	Intervention	Additional instructions provided for participants whilst enrolled in trial	Wash-out-period prior to commencing trial	Technique used to assess microbiota composition in feces	Methods used to report changes in microbiota composition/species
Fernandez-Raudales et al.^39^	Randomized double blinded, 3-arm trial	3 months	NM	Bovine milk	Avoid dietary supplements and antibiotics Inform investigators if became ill during the course of study Avoid fluid milk other than provided by study	One-week washout period. Provided a list of food products to avoid including soy products	Technique 1: qPCR	Bacterial abundance reported in log10 copy number per gram of dry feces
							Technique 2: 16s rRNA sequencing using bTEFAP Region: V1-V3 Pipeline:.NET and # C Database: customed NCBI	Diversity measures: richness (ACE, Chao1) Changes in OTU
Link-Amster et al.^40^	Randomized controlled, 2-arm trial	3 weeks	NM	Fermented milk (yogurt)	No specific additional instructions for participants were reported	Two-week washout period in which all fresh fermented dairy products were excluded	Culture dependant techniques Enterobacteria were cultured on Drigalski agar Lactobacilli were cultured on MRS agar Bifidobacteria were cultured on Eugon agar	Bacterial counts were reported in CFU/g feces
Odamaki et al.^41^	Randomized controlled open, 3-arm parallel trial	19 days	Samples size was not based on power calculation; aimed to enroll maximum numbers due to the lack of previous data	Yogurt	The participants were prohibited to consume oligosaccharides, probiotics, dietary fibers, other yogurt, and alcohol	a seven-day pre-observation period was followed where all participants were instructed to follow their ordinary diet	16s rRNA Region: V3-V4 Pipeline: not mentioned Database: Greengenes	Diversity measures: alpha and beta-diversity Relative abundance of bacterial genera
Odamaki et al.^42^	Randomized open, 2-arm parallel trial	12 weeks	NM	Yogurt	No specific additional instructions for participants were reported	All participants were instructed to participate in a run-in period in which they did not ingest yogurt until notification of their screening results.	qPCR	Bacterial numbers reported in CFU/g wet weight feces
Yilmaz et al.^43^	Randomized controlled open-label, 2-arm trial	4 weeks	NM	Kefir	No specific additional instructions for participants were reported	No information on wash-out period reported	qPCR	Bacterial numbers reported in CFU/g
Beaumount et al.^44^	Randomized controlled double-blind, 3-arm parallel trial	5 weeks	Powered to detect changes in SCFA	Casein powder	No specific additional instructions for participants were reported	Two weeks run-in-period where participants we instructed to consume normal protein diet corresponding to their habitual energy intake	16s rRNA Region: V3-V4 Pipeline: QIIME and Mothur Database: not mentioned	Diversity measures: alpha and beta diversity
Reimer et al.^45^	Randomized controlled, double-blind, 4-arm study	12 weeks	The study was powered to detect change in body composition	Whey bar	Participants were advised to maintain their habitual diet and current level of activity	No information on wash-out period reported	16 s rRNA Region: V3-V4 Pipeline: not mentioned Database: not mentioned	Relative abundance Diversity measures: alpha and beta diversity
Bendtsen et al.^46^	Randomized controlled, 2-arm parallel trial	24 weeks	Powered to detect difference in weight loss and fecal fat excretion	Quantity of dairy consumed	No specific additional instructions for participants were reported	No information on wash-out period reported	16s rDNA Region: V3+ V4 Pipeline: QIIME Database: Greengenes	Relative abundance Diversity measures: alpha and beta diversity

bTEFAP: bacterial tag-encoded FLX amplicon pyrosequencing; ACE: abundance-based coverage estimator; OUT: operational taxonomic unit; qPCR: quantitative polymerase chain reaction; NM: not mentioned.

### The effects of the type of dairy and dairy derivatives on the gut microbiota

#### Milk

Fernandez-Raudales et al.^39^ compared the effect of three-months of bovine milk consumption to two types of soymilk (low glycine soymilk and conventional soymilk) consumption on the gut microbiota composition in obese participants, using qPCR and bTEFAP. The qPCR results demonstrated that there was a significant increase in *Lactobacillus* and a non-significant increase in *Bifidobacterium* copy numbers in the bovine milk group compared to the soymilk groups at the end of the intervention. The bTEFAP showed that bacterial diversity was reduced at the end of intervention in all three groups, independent of the type of milk consumed. Similarly, bacterial richness, estimated using ACE and Chao1 indices, declined in all three groups. In the bovine milk group, the Firmicutes to Bacteroidetes ratio remained unchanged, while *Roseburia* tended to increase and *Prevotella* tended to decrease (Table 4).

**Table 4.** Changes in gut microbiota from feces according to dairy intervention.

			Changes in the fecal microbiota in response to the intervention/comparator			
			Changes of relative abundance in phylum, genus or species level			
	Study	Intervention/comparator	Phylum	Genus	Species	Diversity changes (alpha or beta diversity)
	Fernandez-Raudales et al.^39^	Arm 1: Bovine milk, 500 mL/d	^⊘^Firmicutes Bacteroidetes ↓ Proteobacteria ↑ ^⊘^ Firmicutes/Bacteroidetes	*Roseburia* ↑ *Lactobacillus* ↑ *Prevotella* ↓ *Bifidobacterium* ↑	NR	ACE ↓ Chao1 ↓
		Arm 2: Low Glycine soymilk, 500 mL/d	Firmicutes ↓ Bacteroidetes ↑ Proteobacteria ↑ Firmicutes/Bacteroidetes ↓	*Roseburia* ↓ *Prevotella* ↑	NR	ACE↓ Chao1 ↓
Milk		Arm 3: Conventional soymilk, 500 mL/d	Firmicutes ↓ Bacteroidetes ↑ Proteobacteria ↑ Firmicutes/Bacteroidetes ↓	*Roseburia* ↓ *Prevotella* ↑	NR	ACE ↓ Chao1 ↓
Fermented dairy	Link-Amster et al.^40^	Arm 1: Fermented milk (yogurt), 3 × 125 g units per day	NR	*Lactobacillus* ↑ *Bifidobacterium* ↑	*Lactobacillus acidophilus* ↑	NA
		Arm 2: No fermented dairy/no placebo	NR	^⊘^*Lactobacillus* ^⊘^*Bifidobacterium*	^⊘^*Lactobacillus acidophilus*	NA
	Odamaki et al.^41^	Arm 1: Yogurt provided during two phases of the trial, 200 g/d	NR	^⊘^*Odoribacter* ^⊘^*Bilophila*, *Dorea* ↓ ^⊘^*Ruminococcus* ^⊘^*Bifidobacterium*	NR	No changes in alpha or beta diversity
		Arm 2: Yogurt provided during one phase of the trial (during the balanced diet phase), 200 g/d	NR	*Odoribacter* ↓ *Bilophila*, ↓ *Dorea* ↓ *Ruminococcus* ↓ *Bifidobacterium* ↑	NR	No changes in alpha or beta diversity
		Arm 3: No yogurt during both phases of the trial	NR	*Odoribacter* ↑ *Bilophila*, ↑ *Dorea* ↑ *Ruminococcus* ↑ *Bifidobacterium* ↓	NR	No changes in alpha or beta diversity
	Odamaki et al.^42^	Arm 1: Yogurt, 160 g/d	NR	NR	*Bacteroides fragilis* ↓	NA
		Arm 2: UHT milk, 200 mL/d	NR	NR	^⊘^*Bacteroides fragilis*	NA
	Yilmaz et al.^43^	Arm 1: Kefir 400 mL/d	NR	*Lactobacillus* ↑	NR	NA
		Arm 2: No fermented dairy/no placebo	NR	^⊘^*Lactobacillus*	NR	NA
Dairy derivatives	Beaumount et al.^44^	Arm 1: Casein powder to provide 15% habitual E intake	NR	NR	NR	No changes in alpha or beta diversity
		Arm 2: Maltodextrin powder to provide 15% habitual E intake	NR	NR	NR	No changes in alpha or beta diversity
		Arm 3: Soy powder to provide 15% habitual E intake	NR	NR	NR	No changes in alpha or beta diversity
	Reimer et al.^45^	Arm 1: Whey protein bar (5 g), twice a week	^⊘^Actinobacteria	^⊘^*Bifidobacterium*	NR	No changes in alpha or beta diversity
		Arm 2: Prebiotic bar (5 g), twice a week	Actinobacteria ↑	*Bifidobacterium* ↑	NR	↓alpha diversity (↓ Chao1)
		Arm 3: Whey+prebiotic bar (5 g), twice a week	Actinobacteria ↑	*Bifidobacterium* ↑	NR	↓alpha diversity
		Arm 4: Control- no bars	^⊘^Actinobacteria	^⊘^*Bifidobacterium*	NR	No changes in alpha or beta diversity
Dairy quantity	Bendtsen et al.^46^	Arm 1: High dairy (HD)	No significant taxonomic changes in genus phylum level	No significant taxonomic changes in genus level	NR	No changes in alpha or beta diversity
		Arm 2: Low dairy (LD)	No significant taxonomic changes in genus phylum level	*Veillonella* ↓	NR	No changes in alpha or beta diversity

↑ abundance increased; ↓ abundance decreased; ^⊘^Unchanged, NR: not reported in the study, NA: not applicable to the study.

#### Fermented dairy

Four RCTs assessed the effects of fermented dairy (yogurt and kefir) on the gut microbiota.^40-43^ Of these, three^40-42^ reported the effect of yogurt and one^43^ study reported the effect of kefir on the gut microbiota composition. Link-Amster et al.^40^ reported that consumption of fermented yogurt increased *Lactobacillus* and *Bifidobacterium* counts during the three week intervention period compared to the control arm, which consumed a usual diet but no fermented dairy/placebo. Additionally, a significant four-fold increase in specific serum IgA titer against *Salmonella typhi* Ty21a was observed in the intervention arm.^40^ Odamaki et al.^41^ demonstrated that yogurt intake reversed the shift created in the microbiota composition due to the intake of a mainly animal-based diet. All participants in this trial received a diet based mainly of animal products for five days, followed by a ‘balanced-diet’ for 14 days. Participants were randomized to one of three groups: the first group ingested 200 g of yogurt during both the animal and balanced diet period, while subjects in the second group ingested 200 g yogurt only during the balanced-diet period. Subjects in the third (control) group did not receive yogurt during either of the dietary phases. No differences in the alpha or beta diversity of the fecal microbiota were observed during the study for any groups during or following the dietary interventions.^42^ However, at the end of the animal-based diet phase, the relative abundance of genera *Odoribacter, Bilophila, Dorea*, and *Ruminococcus* increased, and the genus *Bifidobacterium* decreased, in participants who had not yet received the yogurt intervention (i.e. second group ingesting yogurt in the ‘balanced diet’ phase only, and the control group). The relative abundances of these genera were significantly associated with the fat, carbohydrate, and fiber contents of the animal-based diet. Such changes were not observed in those receiving yogurt alongside the animal-based diet, prompting the authors to conclude that the effects of the animal-based diet on gut bacteria were mitigated by the yogurt consumption. In the two intervention groups, the microbiota composition returned to baseline levels following the balanced diet phase, but not in the control group (Table 4). The third study showed that yogurt intake for the period of 8 weeks significantly decreased the cell number of enterotoxigenic *Bacteroides fragilis* compared to the control arm that consumed milk.^42^

The RCT conducted by Yilmaz et al.^43^ investigated the effect of kefir on the gut microbiota in patients with inflammatory bowel disease. The test group consumed 400 mL/d of kefir for 4 weeks while the control group did not consume any fermented dairy (no placebo was provided either). The genera *Lactobacillus* was higher in the test group compared to control group (Table 4) at the end of the study period.

#### Dairy derivatives

Beaumont et al.^44^ randomly assigned healthy overweight participants into three groups to receive a dietary supplement composed of soy protein, casein protein, or maltodextrin for 5 weeks. There were no significant changes in alpha or beta diversity within or between the three groups over the period of intervention.^44^ Another RCT using whey protein reported similar results. Reimer et al.^45^ randomized obese subjects to four groups to receive isocaloric snack bars with different ingredients over a period of 12 weeks: (1) control bar; (2) inulin prebiotic bar; (3) whey protein bar; and (4) a combination whey protein and inulin bar. At the end of the intervention, there were changes in overall microbial structure in group two (inulin bar) and four (combination of whey protein and inulin bar), while the abundance of genus *Bifidobacterium* increased, and alpha diversity also decreased in these two groups (Table 4). In contrast, there were no differences reported in either group one (control bar) or three (whey protein bar).

### The effects of the quantity of dairy consumed on the gut microbiota

In a 24-week RCT, Bendtsen et al.^46^ observed that the quantity of dairy consumed did not result in significant changes to gut microbiota composition (genus or taxa level) in obese subjects. Participants in this trial were randomized to receive either a hypocaloric diet with high dairy intake or hypocaloric diet with low dairy intake. In this study, the quantity of dairy consumed was defined by calcium. The high dairy group consumed dairy in an amount equivalent to 1500 mg/d of calcium and the low dairy group consumed dairy equivalent to 600 mg/d of calcium. There were no significant changes observed in the gut microbiota composition or alpha diversity between the two groups. However, the relative abundance of genus *Veillonella* reduced between baseline and week 24 in the low dairy group (Table 4).^46^

## Discussion

To our knowledge, this is the first systematic literature review to collate evidence on all types of dairy and dairy-derived products and evaluate their possible effects on human gut microbiota composition. Our study results revealed that milk intake increased the relative abundance of genera *Lactobacillus* and *Bifidobacterium* and reduced the bacterial diversity.^39^ Similarly, fermented dairy intake (i.e. yogurt and kefir) increased the abundance of genera *Lactobacillus* and *Bifidobacterium*.^40,41,43^ Moreover, two studies indicated that yogurt consumption was protective against pathogenic bacterial strains i.e. *Bacteroides fragilis* and *Salmonella typhi*.^40,42^ However, it was demonstrated that neither casein or whey nor quantity of dairy consumed prompted changes to the gut bacterial taxa from phylum to species level and overall diversity.^44-46^ Nevertheless, it was indicative that milk and fermented dairy products may modulated the gut microbiota in a manner that may benefit the host by facilitating the growth of *Lactobacillus* and *Bifidobacterium*, which are considered probiotic species that – by definition – benefit host health.^47^

In our study, the changes that dairy products may prompt to the gut microbiota was analyzed in relation to the biological profile and quantity of dairy consumed; this was taking in to consideration the distinct biological profile of each dairy products (i.e. unfermented dairy, fermented dairy, dairy derivatives) and the differing pathways by which they might influence the gut microbiota. Although some dairy products (i.e. milk, yogurt, kefir) demonstrated commonality in enhancing the growth of certain bacterial taxa (i.e. *Lactobacillus, Bifidobacterium*), some studies reported differential effects of dairy products on the gut microbiota. Reduced bacterial diversity – a marker often associated with host health^48,49^ – was reduced in participants consuming bovine milk, as well as in the control conditions, and there were indications of reductions in *Prevotella* in the bovine milk group. Although *Prevotella* is considered a commensal phylotype, some studies have reported a link between certain strains of *Prevotella* and inflammatory status.^50,51^ However, neither the quantity of dairy consumed, nor dairy derivatives such as casein or whey isolates, had a meaningful impact on the human gut microbiota composition. Thus, the impact of dairy consumption on the gut microbiota remains unclear.

### Limitations and strengths of the study

Some of the exclusion criteria that were deployed in designing this study such as excluding studies that are not published in English and excluding abstracts (i.e. no full text available in English, no full text available) may have eliminated eligible studies and this is a limitation (Appendix 1). Only three RCTs were double blinded^39,44,45^ and others were open-label studies.^40-43,46^ The unblinded trials are prone to bias estimates of treatment effects.^52^ However, in RCTs with biological outcomes this is less of an issue. Although only RCTs were included in this systematic literature review, there were methodological inconsistencies and heterogeneity in trial settings, study subjects, microbiota analysis techniques (e.g. 16s RNA, culture-dependant methods), microbial outcomes (relative abundance, alpha, and beta-diversity), sample sizes, and study products; this precluded us from conducting a meta-analysis.

The use of varying microbial analysis techniques is another problematic characteristic of included studies; firstly, the use of culture-dependant analyses in one study^40^ reduced the comparability with other studies, which used culture-independent techniques such as qPCR and 16s rRNA.^39,41-46^ Although five studies used 16s RNA high throughput sequencing, the analysis methods encompassed different variable regions, different pipelines, and different databases that may have influenced the result to varying extents (Table 3).^39,41,44-46^ Moreover, fecal sample collection methods, sample processing methods, and DNA extraction methods influence the output generated.^53-55^ RNA later stabilizer solution was used for sample collection in one study,^41^and others employed cold-chain collection methods (i.e. no stabilizer). Importantly, the use of stabilizer kits (i.e. RNA later) may lead to disruptions in sequencing results, such as decreased species relative abundance.^56,57^ Additionally, studies differed significantly in terms of methods used to extract bacterial DNA, which can influence DNA yield, purity, and ultimately, microbiome outcomes.^58,59^As such, this may have also influenced the results. Additionally, three studies limited the analysis of the gut microbiota *a priori* to certain phylotypes: Firmicutes (*Lactobacillus*) and Actinobacteria (*Bifidobacterium*)^40,43^ or Bacteroidetes (*Bacteroides fragilis*),^42^ making comparisons challenging. Whilst there are emerging methodologies for power calculations of microbiome studies, there is no established practice for microbiome outcomes in RCTs.^60^ Besides, none of the included studies undertook a power calculation for microbiome outcomes, thus it is not possible to establish whether they were adequately powered to detect differences at the various taxonomic levels. This is a limitation of all studies included. Also, all studies were small and given the inherent multiple testing and corresponding false discovery rate corrections required for microbiome analyses it is possible that all included studies were underpowered.

Host characteristics are key in determining the gut microbiota composition and disparities in sex, population type (e.g. healthy, obese or overweight, inflammatory bowel disease) and geographical location differently influence the gut microbiota composition.^22,24-27^ The RCTs that were included in evidence synthesis were conducted in different geographical regions and on different populations (i.e. diseased and healthy), which may have also affected the gut microbiota outcomes. Of the eight studies, six studies^39,40,43-46^ considered a range of external key factors including use of antibiotics, medications, probiotics, and supplements that may also influence the gut microbiota.^61,62^ The impacts from these factors were controlled or omitted by entailing stringent inclusion/exclusion criteria (e.g. excluding participants with antibiotics use in the past three months) (Table 2). Moreover, two^39,41^ studies monitored conditions (e.g. illness, use antibiotic, diet) that may impact the gut microbiota whilst participants were engaged in the intervention. All but three studies^43,45,46^ had a washout/run-in-period that preceded the intervention phase, which ensured dietary normalization and exclusion of any residual impacts from dairy prior to baseline; this is important given that the interventions comprised dairy products (Table 3). However, the RCTs included in this review did not assess compliance to the intervention/study products in both the experimental and control groups; consequently, noncompliance (i.e. consuming dairy external to the intervention) may be unaccounted and this is a limitation of all studies.

Components of dairy (e.g. protein, fat) and type of dairy (e.g. fermented and unfermented) may influence the gut microbiota differently; therefore, it is important to stipulate detailed description of the dairy product composition used in the intervention (e.g. low fat milk or full cream milk) in order to assess its impact on the gut microbiota composition. Two studies did not provide details on dairy composition^39^ or the type of dairy^46^ used as intervention, which precluded meaningful interpretations.

Of note, four studies^40-43^ assessed the impact of fermented dairy products that were produced by inoculating probiotic cultures; this also makes the generalization of conclusions challenging as different cultures are likely to have differential impacts on the gut microbiota. Moreover, during the process of fermentation, a myriad of biologically active molecules are synthesized including bio-active peptides, vitamins, and prebiotics, adding more functional and nutritive value to the original substrate, which in turn may influence the gut microbiota differentially.^63^ When assessing the impacts of fermented dairy on the gut microbiota in humans, distinguishing the effects exerted by dairy components versus bacterial cultures is not possible due to synergistic effects.

Finally, all studies included in this systematic review used stool samples as a proxy to determine the gut microbiota composition. The microbiota in the stool sample may not reflect the species in the whole gastrointestinal (GI) tract, because stools mostly represent the bacterial species in the lumen and the lower GI tract.^23,64^ Thus, stool samples may not reflect the change to the bacterial species in the upper GI tract in response to external (e.g. diet, medications) or internal (e.g. disease) determinants.

### Mechanisms by which dairy may influence the gut microbiota

**Figure 2.**
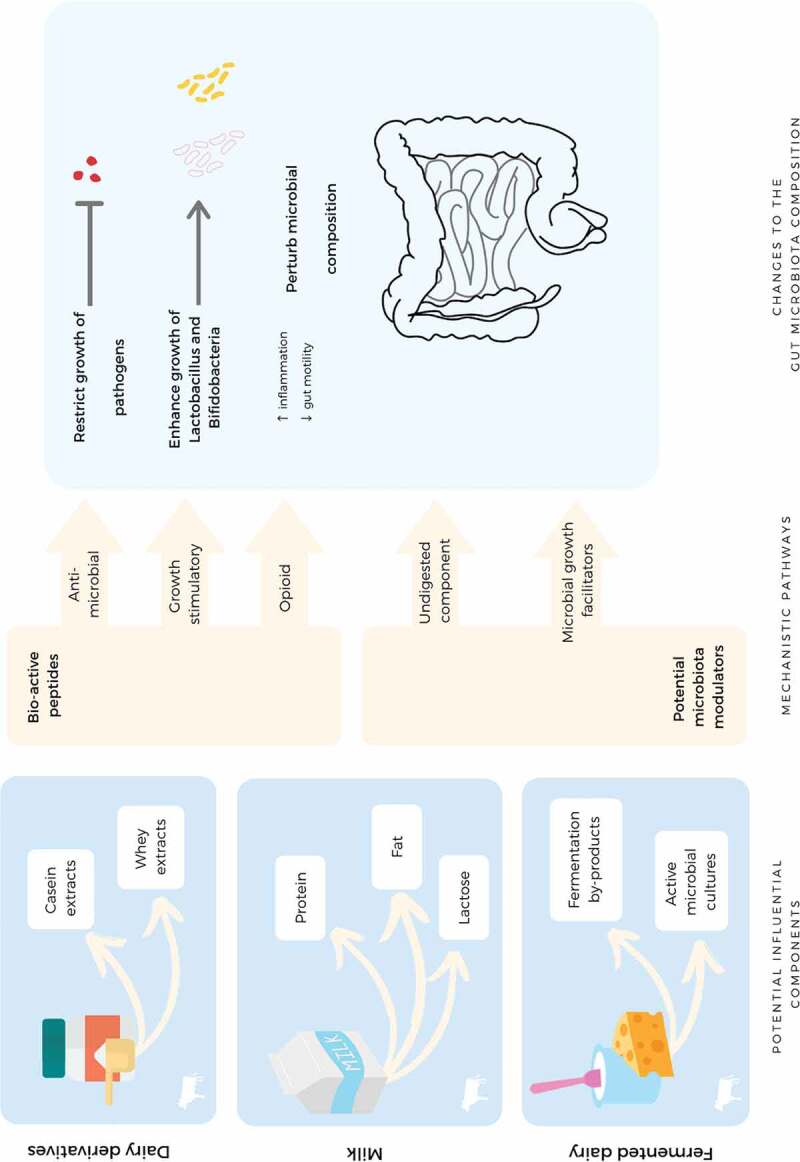
Mechanistic pathways by which dairy and its components may impact the gastrointestinal environment and the gut microbiota composition.

The gut microbiota is constructed of five primary bacterial phyla (Firmicutes, Bacteroidetes, Actinobacteria, Proteobacteria and Fusobacteria), although Firmicutes and Bacteroidetes comprise 90% of the gut microbiome.^65^ However, this proportion may not be similar in all individuals due to inter-personal variations created by environment, genetics, diet, and disease.^22,24-27,66^ Diet is increasingly recognized as a modulator of the gut microbiota.^21^ Dairy products contain an array of nutrients including proteins, lipids, carbohydrates, amino acids, minerals and vitamins; any of these components may have potential to influence the gut microbiota composition.^36,67,68^ However, we speculate that dairy products may influence the gut microbiota via mechanistic pathways such as: (1) facilitating the growth of beneficial strains,^69^ (2) suppressing the growth of pathogenic strains,^70^ and (3) altering the gastrointestinal (GI) environment (Figure 2).^33,71,72^

Antimicrobial and stimulatory peptides produced by milk protein digestion and lactose facilitates the growth of so-called beneficial bacterial genera such as *Lactobacillus* and *Bifidobacterium*. Milk fat and opioid peptides (produced during protein digestion) negatively alter the luminal environment by inducing inflammation and increasing the gastrointestinal transit time, which consequently perturb the gut microbiota composition. Fermented dairy products favor the growth of beneficial bacteria in the gut via the active bacterial cultures and fermentation by-products.

### Promoting the growth of beneficial bacteria

Five studies^39-43^ included in this review demonstrated the potential of dairy products including milk and fermented dairy (i.e. yogurt and kefir) to increase the growth of so-called beneficial bacteria.^40-43^ *Lactobacillus* and *Bifidobacterium* are two major bacterial genera that are considered to be ‘probiotics’ due to their demonstrated benefits to host health, such as improving metabolic disequilibrium, immune modulation and regulating inflammation.^73^ Components of dairy such as lactose and protein possess potential to stimulate the growth of these bacterial genera. Lactose is the key milk carbohydrate; it exists in a concentration of 53 g/L and is often referred to as ‘milk sugar’.^74^ Both *in vitro* and *in vivo* studies have demonstrated that lactose increased the growth of *Bifidobacterium* and *Lactobacillus*.^67,75^ Lactose also contains a prebiotic index of 5.75, which is similar to many other prebiotics.^69,76^ Prebiotics are a fermentable ingredient that instigates specific changes to both the composition and/or activity of the gastrointestinal microbes rendering health benefits. Milk proteins also hold potential to facilitate the growth of *Bifidobacterium and Lactobacillus*. Casein forms 80% of milk protein and is composed of four sub-units (α-caseinS1, α-caseinS2, β-casein, κ-casein). A κ-casein derived hydrolyzates demonstrated growth stimulatory effects on *Bifidobacterium bifidum* in a synthetic culture media.^68^ Whey protein forms 20% of milk proteins and include the water-soluble protein fraction: α-lactoalbumin, β-lactoglobulin, immunoglobulins (Ig), lactoferrin, serum albumin, lactoperoxidase and lysozymes. Short peptides produced by the proteolytic digestion of β-lactoglobulin showed growth proliferation effects on *Bifidobacterium and Lactobacillus spp*.^77^ Further, lactoferrin hydrolyzates increased the growth of *Bifidobacterium adolescentis* B-1 in a dose dependant manner.^78^

### Suppressing the growth of pathogens

Two studies that were included in this review demonstrated the potential of dairy products to retard the growth of pathogenic strains.^40,42^ The study by Odamaki et al.^42^ showed that consumption of yogurt reduced *Bacteroides fragilis*, a strain that is associated with diarrheal disease, inflammatory bowel disease and colorectal cancer.^79,80^ Link-Amster et al.^40^ reported that consumption of fermented yogurt increased specific serum IgA titer against an enteropathogenic strain, *Salmonella typhi*. Dairy products may suppress the growth of pathogenic strains via two pathways: (1) competitive exclusion of pathogens by facilitating the growth of specific bacterial strains; and (2) yielding bio-active peptides (antimicrobial peptides) that retard the growth of pathogenic strains.

As discussed previously, certain components of dairy itself (e.g. lactose, bio-active molecules produced by fermentation and peptides) facilitate the growth of probiotic bacteria, which in turn aids in pathogen elimination via creating competition for adhesion sites and producing anti-microbial peptides.^47^ When subjected to enzymatic digestion, milk proteins can yield bio-active peptides that contain potential antimicrobial properties. Isracidin, a peptide that is produced by enzymatic digestion of α-caseinS1, demonstrates a broad spectrum of antibacterial action by restricting the growth of pathogenic strains such as *Staphylococcus aureus, Streptococcus pyogenes* and *Listeria monocytogenes*.^70^ Casocidin is another antimicrobial peptide that is produced by the enzymatic digestion of α-caseinS2 and demonstrates potential to inhibit *Escherichia coli* and *Staphylococcus carnosus*.^70^ One animal study showed that rats fed a casein-prebiotic mix had lower numbers of *Clostridium perfringens* compared to those fed a soy-prebiotic mix.^34^ Additionally, whey protein yields antimicrobial peptides and effectively arrests the growth of *Escherichia coli, Klebsiella pneumoniae, Staphylococcus aureus, Staphylococcus epidermidis, Staphylococci* and *Candida albicans*.^81,82^

### Altering the gastrointestinal environment

Dairy components, in particular milk proteins and fats, are capable of changing the luminal environment and thereby instigating changes to the gut microbiota composition.^33,71,72^ The A1 beta-casein protein fraction in dairy has shown to alter the GI transit time and trigger GI inflammation by producing opioid peptides, which in turn may influence the gut microbiota composition.^83-85^ Although evidence do exist to show the potential of A1 beta-casein to increase GI transit time and trigger inflammation, the subsequent impact of these events on the gut microbiota has not been studied yet in both animal and human studies.^86-92^ Our study did include research that assessed the impact of casein extracts on the gut microbiota,^44^ however, revealed no impact on the gut microbiota. Evidence from animal models demonstrates that milk fat intake reduced the abundance of certain bacterial taxa (i.e. *Tenericutes*) with a concomitant elevation in inflammatory markers.^33^ However, in our systematic review most of the studies failed to provide the nutritional composition of dairy products, limiting the understanding of the specific contribution of dairy fats toward the changes in the gut microbiota composition.

## Conclusion

The results of this systematic review suggest that some types of dairy modulate the gut microbiota composition in a manner that may benefit the host. Milk, yogurt and kefir consumption appeared to facilitate the growth of functional phylotypes i.e. *Lactobacillus, Bifidobacterium* that have been related to improved host health. However, the quantity of dairy and dairy derivatives (i.e. casein and whey) revealed negligible impacts on the gut microbiota. Given the heterogeneity in study methods and outcome reporting, and the unblinded nature of trials, it is difficult to draw robust conclusions. Future trials may benefit from encompassing rigorous blinded study design and *a priori* power calculation that ensures adequate sample size. To date the extant evidence on dairy and its implication on the gut microbiota is limited, relative to the widespread application and the different types of dairy available for consumers. Moreover, the impacts of dairy products on the gut microbiota in relation to dairy composition (e.g. full fat, low fat) are understudied. Therefore, further studies are warranted to better understand the broader impacts of dairy products on the gut microbiota.

## Appendix 1

**Table:**

	Record numbers
**Total articles from search**	6592
**Duplicates removed**	1499
**Remaining records for screening**	5093
**Title and abstract exclusion**	
*Animal/in vitro studies*	118
*Studies in children*	15
*Conference abstracts*	11
*Studies on other cattle’s product (e.g. donkey, sheep, goat)*	68
*Studies on human milk*	116
*Studies on infants and infant formulae*	69
*Not in English*	2
*Off topic*	4598
*Review articles*	44
*Not RCTs*	22
**Total ineligible studies**	5063
**Records available for full text assessment**	30
**Full text assessment**	
*Full text not available or not in English*	5
*Comparator not complying with study criteria*	14
*Wrong study*	3
Total ineligible studies	22
**Full text included in analysis**	8
